# Supplementary material for: Creating superconductivity in WB2 through pressure-induced metastable planar defects
Source: Nat Commun. 2022 Dec 22;13:7901. doi: 10.1038/s41467-022-35191-8 (PMC9780245; doi:10.1038/s41467-022-35191-8)
Supplement: Supplementary file 1 — Supplementary Information [file 41467_2022_35191_MOESM1_ESM.pdf]

Supplemental material: Creating superconductivity in WB_2 through pressure-induced metastable planar defects

J. Lim,[1,*] A. C. Hire,[2,3,*] Y. Quan,[1,2,3] J. S. Kim,[1] S. R. Xie,[2,3] S. Sinha,[1] R. S. Kumar,[4] D. Popov,[5] C. Park,[5] R. J. Hemley,[4] Y. K. Vohra,[6] J. J. Hamlin,[1,†] R. G. Hennig,[2,3] P. J. Hirschfeld,[1] and G. R. Stewart[1]

[1]*Department of Physics, University of Florida, Gainesville, Florida 32611, USA*
[2]*Department of Materials Science and Engineering, University of Florida, Gainesville, Florida 32611, USA*
[3]*Quantum Theory Project, University of Florida, Gainesville, Florida 32611, USA*
[4]*Department of Physcis, Chemistry, and Earth and Environmental Sciences, University of Illinois Chicago, Chicago, Illinois 60607, USA*
[5]*HPCAT, X-ray Science Division, Argonne National Laboratory, Argonne, Illinois 60439, USA*
[6]*Department of Physics, University of Alabama at Birmingham, Birmingham, Alabama 35294, USA*

XRD OF WB_2 HR6 PHASE

The XRD peaks of the WB_2 hP12 and hR6 phases are quite similar, such that the broadening of the experimentally measured XRD peaks at high pressures leads to difficulty in conclusively identifying the stable phase. To check whether a strained lattice of the hR6 phase can account for the experimentally observed high-pressure XRD pattern, we perform the following analysis. We strain the DFT relaxed lattice parameters of the hR6 phase and compare the resulting theoretical XRD pattern against the experimental data. The c lattice parameter was kept unchanged from its DFT value because the experimental XRD peaks that contain contributions from the planes perpendicular to the c-axis match almost perfectly with the theoretical peaks. We performed this analysis at two pressures, 85 GPa and 145 GPa, as shown in Figs.1 and 2. In these figures, the peaks denoted by the star symbol are due to the Re-gasket (cyan) and the Ne pressure-transmitting medium (orange). We observe that the strained hR6 lattice fails to produce the experimentally observed pattern at 85 GPa and 145 GPa. This failure of the strained lattice reveals the absence of significant amounts of the hR6 phase. We further rule out the presence of the hR6 phase with the help of the theoretically calculated critical temperatures. According to our calculations, the hR6 phase has a critical temperature of less than 5 K up to 100 GPa, which is far less than the observed T_c of 17 K at 90 GPa.

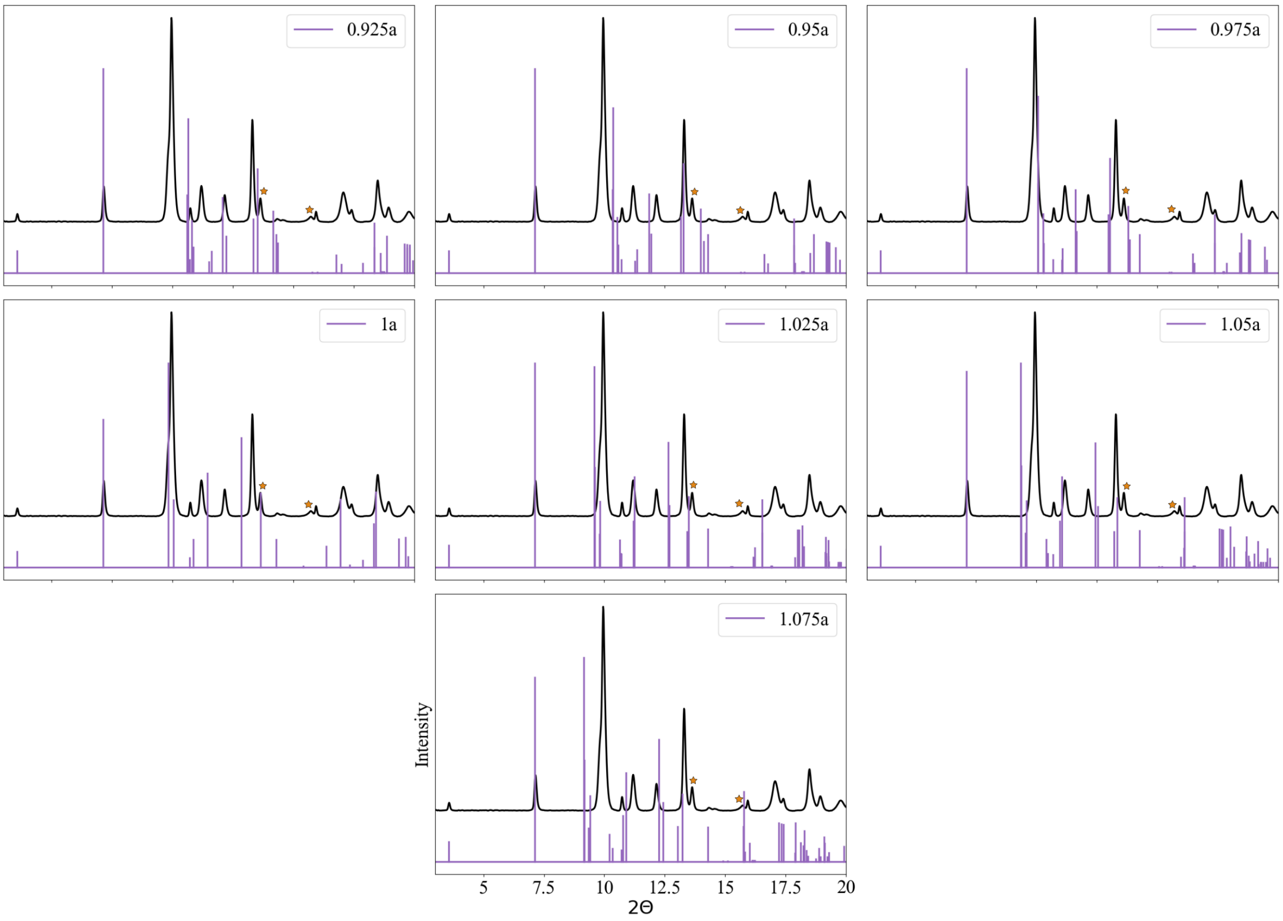

Supplementary Figure 1: Experimentally measured XRD pattern of WB_2 and theoretically calculated peaks of the strained hR6 phase at 85 GPa. The a lattice vector of the structure was strained whereas the c lattice vector was kept unchanged. The legend in each plot indicates the amount by which the a lattice parameter was changed with respect to the DFT relaxed structure.

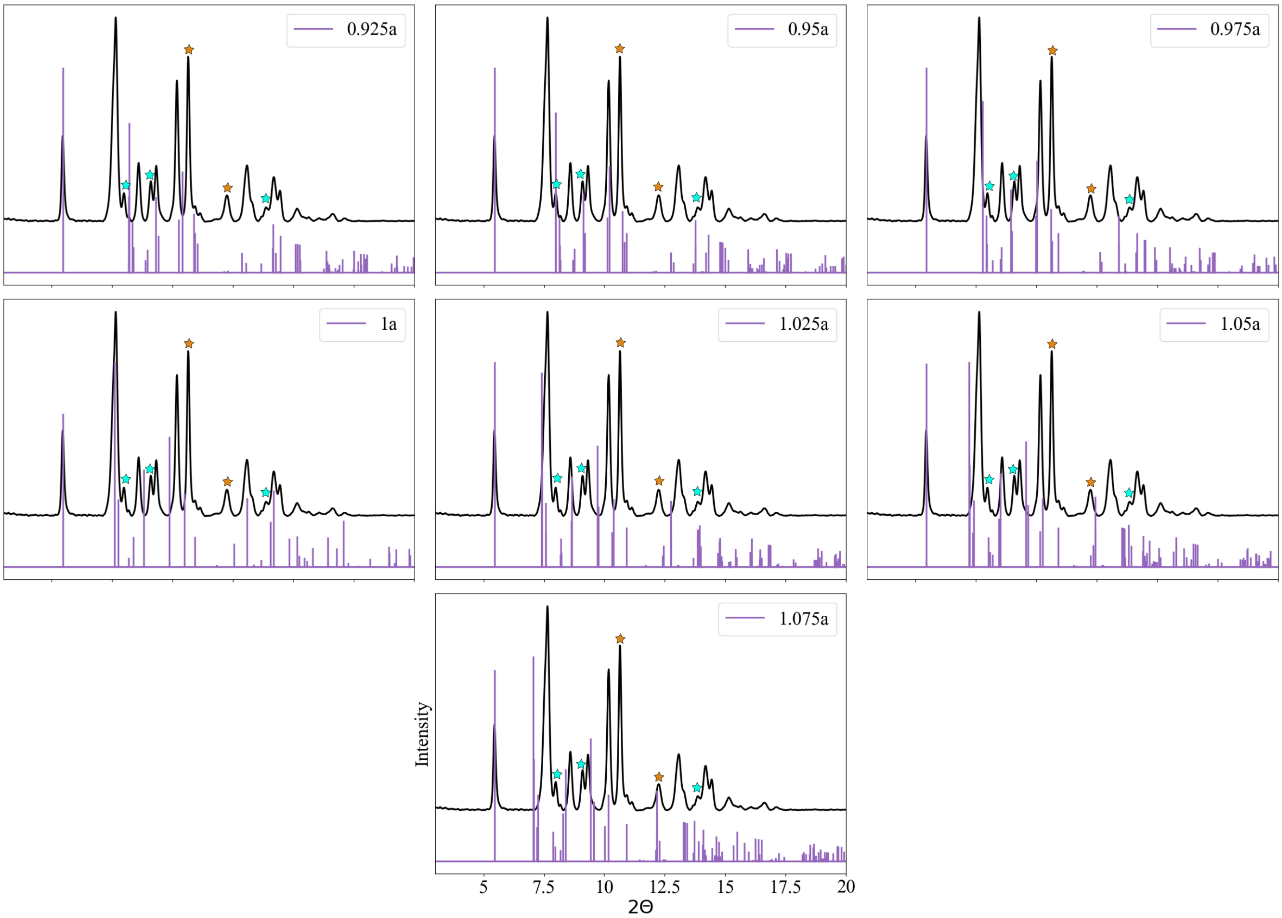

Supplementary Figure 2: Experimentally measured XRD pattern of WB_2 and theoretically calculated peaks of the strained hR6 phase at 145 GPa. The ‘a’ lattice vector of the structure was strained whereas the ‘c’ lattice vector was kept unchanged. The legend in each plot indicates the amount by which the ‘a’ lattice parameter was changed with respect to the DFT relaxed structure.

WB_2 STACKING FAULT AND TWIN BOUNDARY

As the pressure applied to the WB_2 samples increases, stacking faults and twin boundary defects can form inside the sample because of mechanical deformation. Here, with the help of Figures 3 and 4, we present how simulation cells containing these planar defects can be created in the WB_2 hP12 phase by sliding W and B planes. Figures 3(a) and 4(a) show the perfect hP12 unit cells. A cell with a stacking fault is created by sliding the appropriate W and B planes along the $[1/3, 2/3, 0]$ vector in the hP12 cell, as shown in Figure 3(b). In Figure 4(b), the simulation cell of the twin boundary is formed by sliding only two successive W and B planes along the $[1/3, 2/3, 0]$ vector. For calculating the defect formation energy depicted in Figure 4(i), we use simulation cells equivalent to four hP12 unit cells.

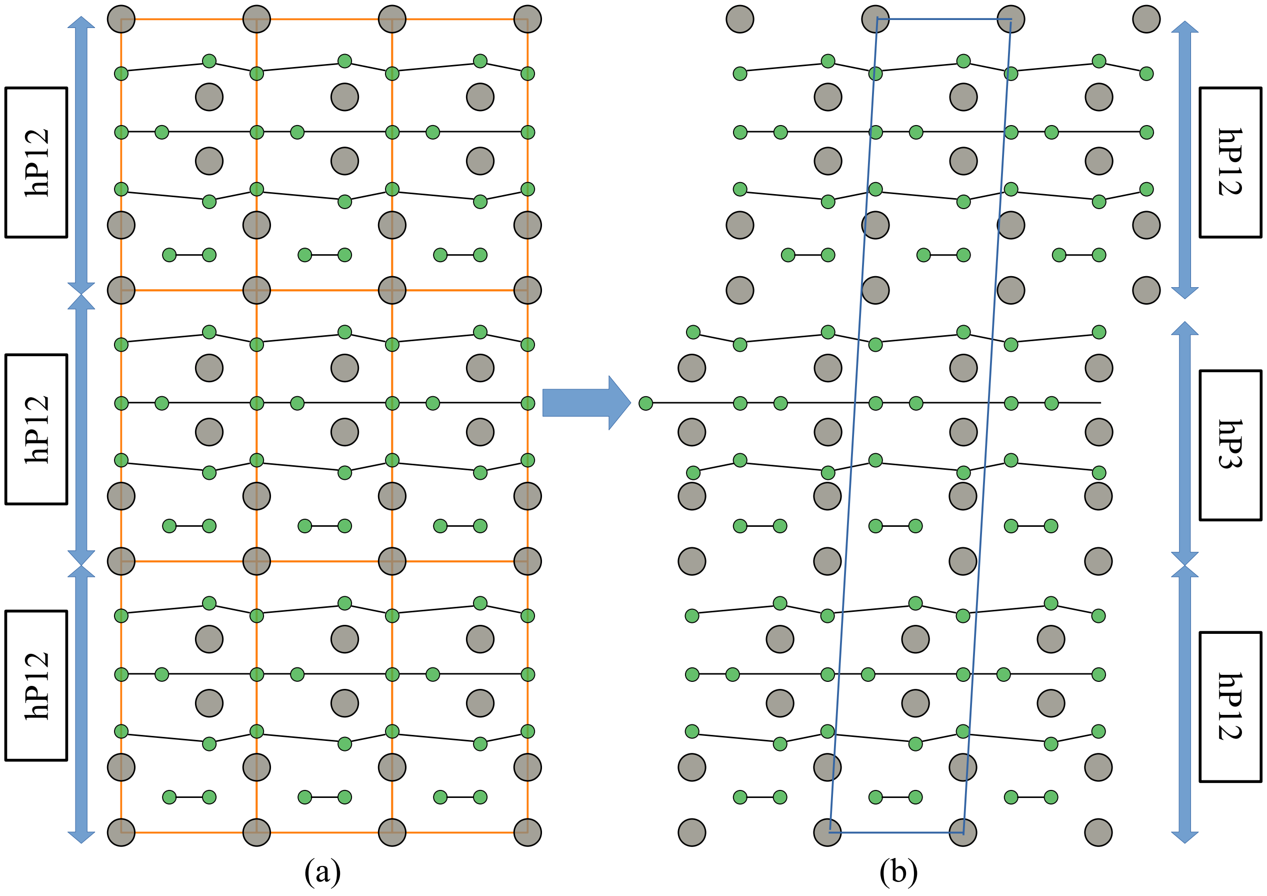

Supplementary Figure 3: Formation of stacking fault by sliding of planes. We expect that the buckled Boron layers in the hP3 region will unbuckle and shift by 1/3 of the ‘a’ lattice vector to lower the energy of the defect structure.

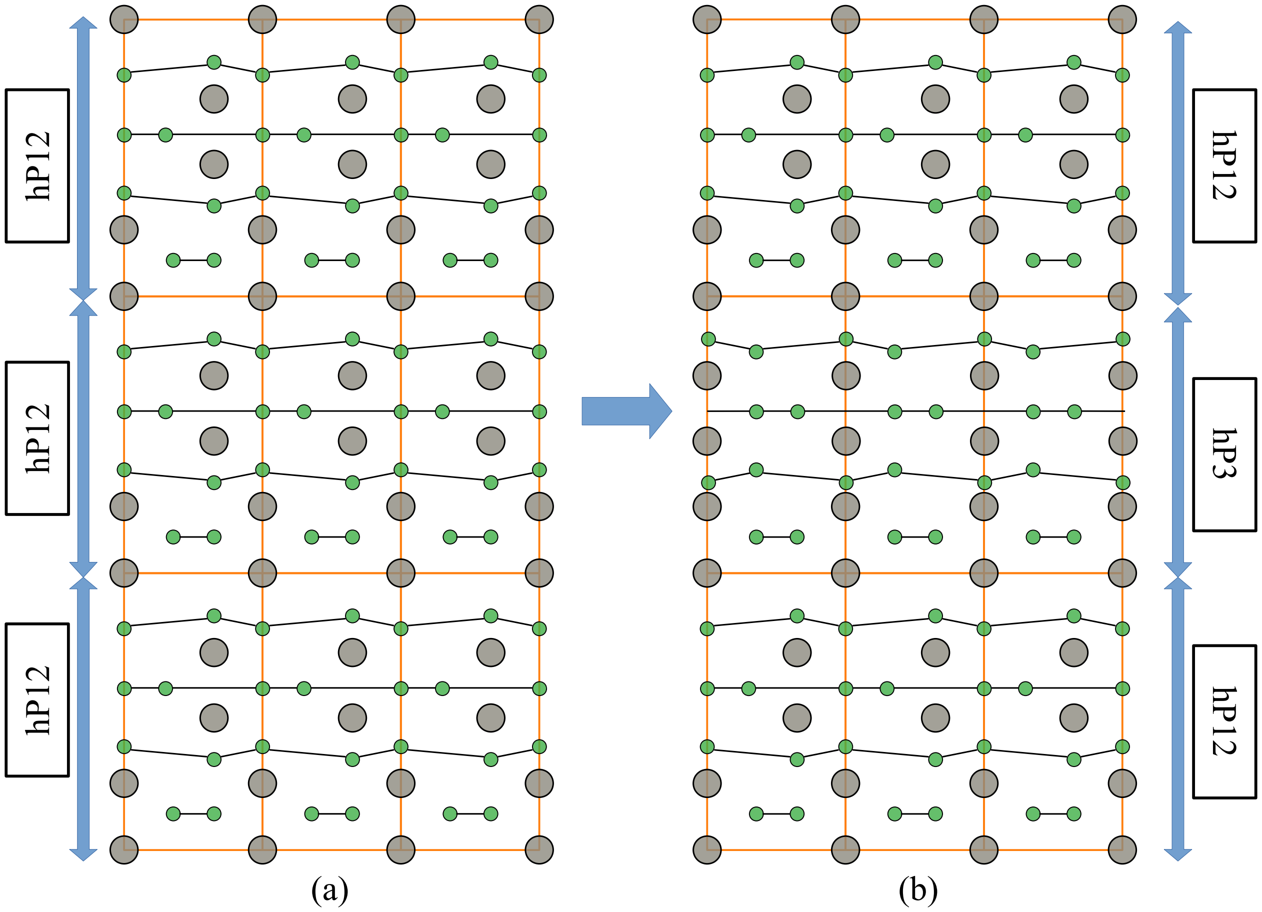

Supplementary Figure 4: Formation of twin boundary by sliding of planes. We expect that the buckled Boron layers in the hP3 region will unbuckle and shift by 1/3 of the ‘a’ lattice vector to lower the energy of the defect structure.

ELECTRONIC STRUCTURE OF WB_2 UNDER PRESSURE

The electronic structures of WB_2 in the experimental space group P6_3/mmc under 0 and 100 GPa are shown in Fig. 5. The states near the Fermi level are mostly tungsten d states and they are important in understanding the superconductivity of WB_2. Pressure usually has the effect of broadening bandwidth. However, the broadening is not uniform. For example, the band that touches the Fermi level at H point under 0 GPa moves to 0.2 eV below the Fermi level under 100 GPa, while the two bands that merge at A point remain 0.3 eV below the Fermi level. Overall, the density of states evolve smoothly with pressure and the Fermi level falls inside a DOS valley, contrary to the sudden emergence of superconductivity at 50 GPa observed in experiments. In addition, the DFT-estimated electron-phonon coupling of WB_2 in the P6_3/mmc phase is small and cannot account for the experimental T_c, which suggests that the superconductivity might not be due to the P6_3/mmc phase. On the other hand, the electron-phonon coupling of WB_2 in the MgB_2 structure is strong, pointing to possible existence of local MgB_2-like structures in certain regions of the experimental sample that is responsible for the superconductivity of WB_2 under pressure.

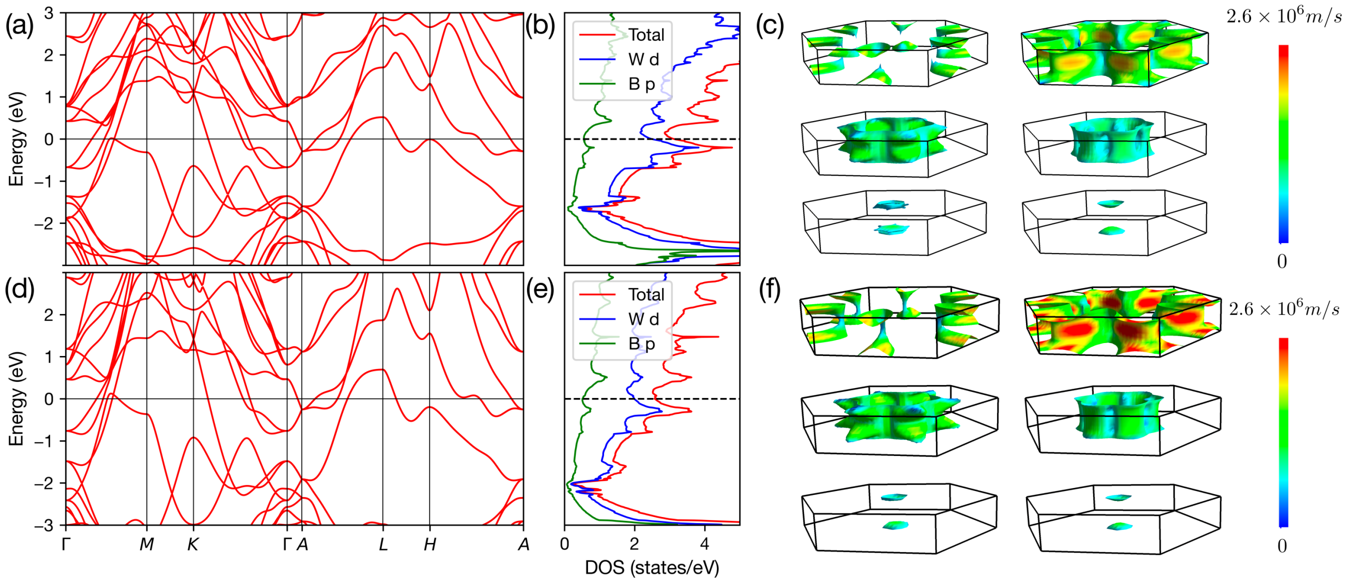

Supplementary Figure 5: Band structures, density of states and Fermi surfaces of WB_2 in P6_3/mmc structure under 0 and 100 GPa. (a) to (c) correspond to WB_2 under 0 GPa and (d) to (f) correspond to WB_2 under 100 GPa.

PRESSURE DEPENDENCE OF PHONON DISPERSION OF WB_2 IN THE HP_3 PHASE

In recent years, high pressure experiments have played an important role in expanding the frontier of phases of materials that can be stabilized. Structures that are otherwise chemically or dynamically unstable under the ambient condition can be easily pressurized into existence. In this section, we present the pressure dependence of the phonon dispersion of WB_2, see 6. The phonon spectrum is calculated by running a QE calculation on a coarse mesh first and then an EPW calculation is carried out to interpolated from the coarse k- and q-meshes to much finer meshes. We have assumed harmonic approximation which can already provide important guidance for understanding the experimental data. Anharmonic effects might have some impact on the exact pressure at which the hP_3 phase becomes unstable. However, the issue of anharmonicity is out of the scope of our current manuscript.

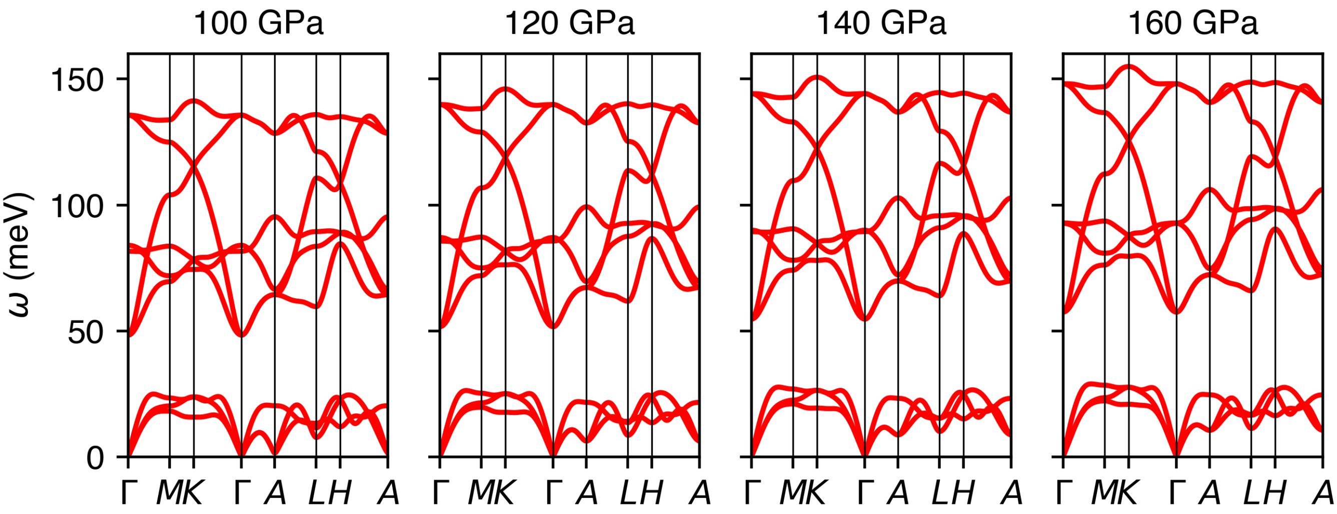

Supplementary Figure 6: Pressure dependence of the phonon dispersion of WB_2 in the hP_3 phase.

TEMPERATURE-DEPENDENT ELECTRICAL RESISTIVITY OF WB_2 UNDER DECOMPRESSION IN RUN 2

Figure 7 shows the decompression behavior of temperature-dependent electrical resistivity of WB_2 in Run 2. The superconducting temperature T_c (90%) at 63 GPa (black curve, first compression) is ∼10 K in consistent with Run 1 as shown in Fig. 1d. When lowering the pressure down to 48 GPa (red curve), interestingly T_c rather increases to ∼13 K and starts to decrease to ∼3 K at further lowering to 4.9 GPa, which shows the irreversible behavior of $T_c(P)$ under decompression. This shows that the superconducting phase of WB_2 is metastable at low pressure.

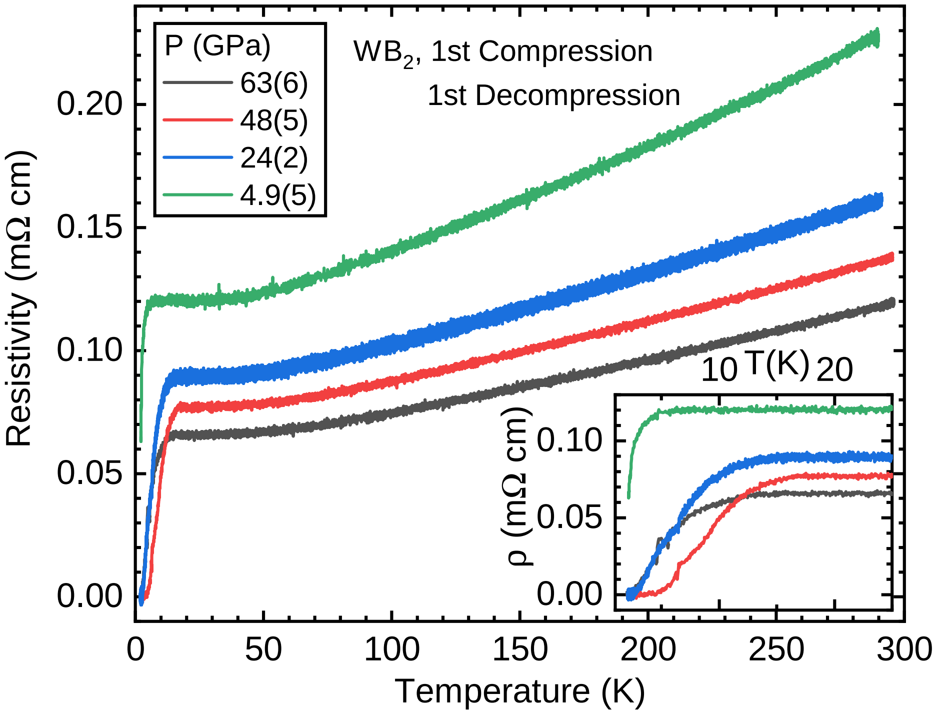

Supplementary Figure 7: Temperature-dependent electrical resistivity curves of WB_2 during decompression from 63 down to 4.9 GPa in Run 2.

TEMPERATURE-DEPENDENT ELECTRICAL RESISTIVITY OF WB_2 UNDER THE SECOND COMPRESSION IN RUN 2

Figure 8 shows the second compression behavior of temperature-dependent electrical resistivity of WB_2 after lowering to ambient pressure (opening the DAC) from the first decompression (Fig. S7) in Run 2. The superconductivity begins to appear from 53 GPa (purple curve) with $T_c \sim 4$ K in consistent with Run 1 as shown in Fig. 1d. The results indicate that the planar defects or twin boundaries responsible for the superconductivity is only (meta)stable under high pressure.

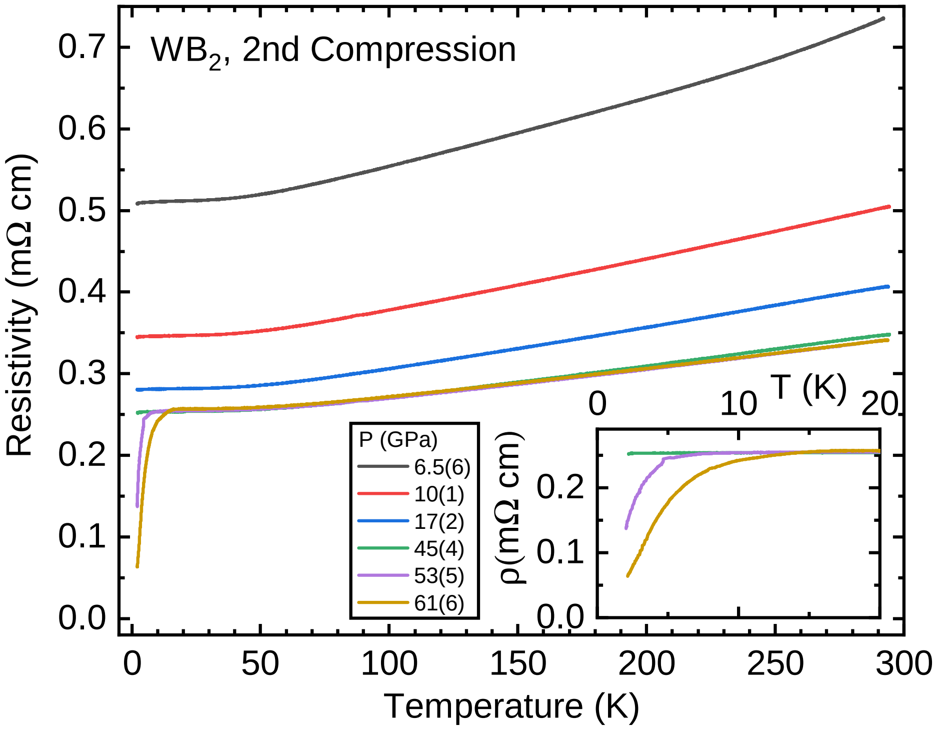

Supplementary Figure 8: Temperature-dependent electrical resistivity curves of WB_2 during the second compression from 6.5 up to 61 GPa in Run 2.

HIGH-PRESSURE XRD PATTERNS OF WB_2 UNDER NONHYDROSTATIC PRESSURE CONDITION

Figure 9 shows high-pressure XRD measurements under nonhydrostatic pressure condition to 98 GPa. Without using any pressure medium, the pressure chamber is filled with the WB_2 samples in these measurements. The XRD patterns in Fig. 9(a) match with hP12 structure (orange line, calculated at 0 GPa) except for few minor additional peaks appearing above 50 GPa that could be assigned to either hP3 or hR6 phase as possible local defects. However, other large peaks associated with the hP3 and hR6 structures (e.g. peaks near 7-8 degrees) are entirely missing from the experimental data. Figure 9(b) shows the XRD pattern at 98 GPa compared to those of the five competing phases in in Fig. 3 of the main text. The data are not not of sufficient quality to permit a full quantitative Rietveld refinement, but nonetheless, the results confirm that the bulk WB_2 sample still remains predominantly in hP12 phase up to 98 GPa under non-hydrostatic conditions. The results are consistent with the quasi-hydrostatic XRD measurements where Ne was used as the pressure medium (Fig. 2, main text).

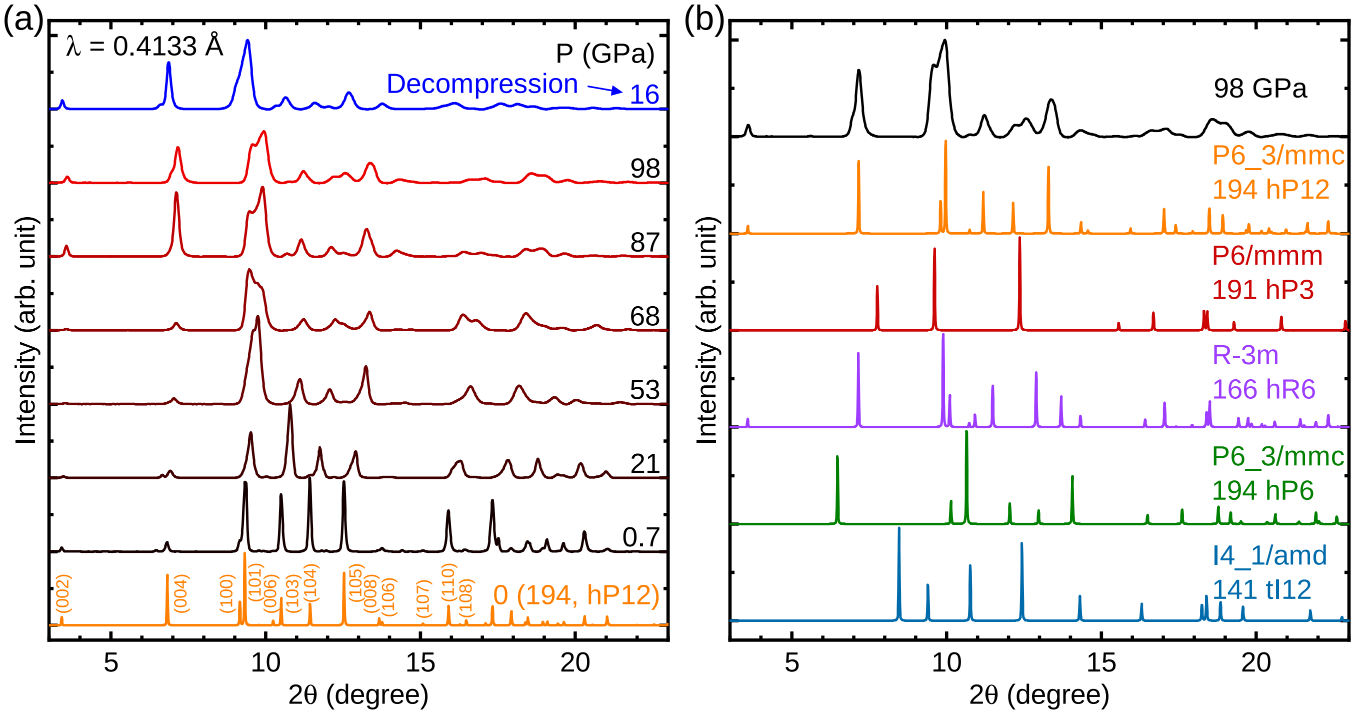

Supplementary Figure 9: (a) High pressure XRD patterns of WB_2 to 98 GPa under nonhydrostatic condition (no pressure medium) (b) Comparison of the XRD pattern at 98 GPa to those of five competing phases.

* These two authors contributed equally to this work.
† Electronic address: jhamlin@ufl.edu
